# The importance of choice in the rollout of ARV-based prevention to user groups in Kenya and South Africa: a qualitative study

**DOI:** 10.7448/IAS.17.3.19157

**Published:** 2014-09-08

**Authors:** Natasha Mack, Emily M Evens, Elizabeth E Tolley, Kate Brelsford, Caroline Mackenzie, Cecilia Milford, Jennifer A Smit, Joshua Kimani

**Affiliations:** 1 Social and Behavioral Health Sciences, FHI 360, Durham, NC, USA; 2 Health Services Research, FHI 360, Durham, NC, USA; 3 Duke Clinical Research Institute, Duke University, Durham, NC, USA; 4 Applied Research Unit, FHI 360, Nairobi, Kenya; 5 MatCH (Maternal, Adolescent and Child Health), Durban, South Africa; 6 Kenya AIDS Control Project, Nairobi, Kenya

**Keywords:** pre-exposure prophylaxis, sub-Saharan Africa, women, ARV-based HIV prevention methods, HIV prevention

## Abstract

**Introduction:**

Stakeholders continue to discuss the appropriateness of antiretroviral-based pre-exposure prophylaxis (PrEP) for HIV prevention among sub-Saharan African and other women. In particular, women need formulations they can adhere to given that effectiveness has been found to correlate with adherence. Evidence from family planning shows that contraceptive use, continuation and adherence may be increased by expanding choices. To explore the potential role of choice in women's use of HIV prevention methods, we conducted a secondary analysis of research with female sex workers (FSWs) and men and women in serodiscordant couples (SDCs) in Kenya, and adolescent and young women in South Africa. Our objective here is to present their interest in and preferences for PrEP formulations – pills, gel and injectable.

**Methods:**

In this qualitative study, in Kenya we conducted three focus groups with FSWs, and three with SDCs. In South Africa, we conducted two focus groups with adolescent girls, and two with young women. All focus groups were audio-recorded, transcribed and translated into English as needed. We structurally and thematically coded transcripts using a codebook and QSR NVivo 9.0; generated code reports; and conducted inductive thematic analysis to identify major trends and themes.

**Results:**

All groups expressed strong interest in PrEP products. In Kenya, FSWs said the products might help them earn more money, because they would feel safer accepting more clients or having sex without condoms for a higher price. SDCs said the products might replace condoms and reanimate couples’ sex lives. Most sex workers and SDCs preferred an injectable because it would last longer, required little intervention and was private. In South Africa, adolescent girls believed it would be possible to obtain the products more privately than condoms. Young women were excited about PrEP but concerned about interactions with alcohol and drug use, which often precede sex. Adolescents did not prefer a particular formulation but noted benefits and limitations of each; young women's preferences also varied.

**Conclusions:**

The circumstances and preferences of sub-Saharan African women are likely to vary within and across groups and to change over time, highlighting the importance of choice in HIV prevention methods.

## Background

Sub-Saharan African women at risk of HIV infection are a heterogeneous group. They include married and unmarried women, women whose partner(s) may have other partners, women in serodiscordant couples (SDCs), women in polygamous relationships, adolescent girls and women who engage in a range of transactional sexual behaviours with one or more partners. Given the diverse characteristics of African women, their needs and preferences for HIV prevention methods are likely to differ.

Stakeholders in HIV prevention continue to discuss the appropriateness of different formulations of antiretrovirals (ARVs) as pre-exposure prophylaxis (PrEP) for HIV prevention among women in sub-Saharan Africa and elsewhere [1]. In particular, these women and others at risk of HIV need formulations they can adhere to given that effectiveness correlates with adherence [2]. This was demonstrated in the iPrex study, which found high effectiveness of oral PrEP among participants with high adherence [3], as well as in the FEM-PrEP and MTN 003 trials, both of which were stopped for futility and later reported low adherence to oral PrEP among participants [4–6]. The CAPRISA 004 trial of 1% tenofovir gel also found that the product's effectiveness corresponded with degree of adherence [7].

Evidence from family planning research provides an insight into how to encourage uptake of and adherence to prevention methods, thereby increasing overall coverage at the population level. Specifically, contraceptive use, continuation and sustained adherence may be increased by expanding the method mix, improving features of methods and increasing the quality of and access to family planning services [8]. Providers’ assistance with the selection and continued use of a method has also been found to be essential to sustained contraceptive use. Another important lesson is the key role of choice in uptake of and adherence to contraception – choice in terms of the user's ability to select a method, a range of contraceptive methods to choose from and places to access them [8–10].

Extrapolating from family planning, to explore the potential role of choice in women's use of HIV prevention methods, we conducted a secondary analysis of data from qualitative research with potential user groups of ARV-based PrEP formulations in Kenya (female sex workers [FSWs], men and women in SDCs) and in South Africa (adolescent and young women). Our objective here is to present our analysis of these groups’ attitudes towards ARV-based HIV prevention, including acceptability, interest and concerns, and their preferences for different formulations – choosing from pills, gel and an injectable.

## Methods

This study was conducted in Kenya and South Africa from 2011 to 2012 in collaboration with local partners. This was subsequent to the 2010 announcement of the positive efficacy results of the CAPRISA 004 clinical trial on 1% tenofovir gel [7] and the iPrex oral PrEP trial [3], but prior to the early closure of the FEM-PrEP oral PrEP trial and the MTN 003 results, which found that oral PrEP (FEM-PrEP and MTN 003) and gel (MTN 003) were not effective because women did not adhere to the products [4–6]. In Kenya, the research was managed by the FHI 360 Kenya office in collaboration with the University of Manitoba/University of Nairobi Sex Work Outreach Program (SWOP), with data collected in Nairobi and Nakuru. In South Africa, data were collected in the eThekwini District of KwaZulu-Natal by MatCH (Maternal, Adolescent and Child Health; Department of Obstetrics and Gynecology; University of Witwatersrand).

Potential participants for focus group discussions (FGDs) with FSWs were identified through the SWOP clinics, a group of Nairobi, Kenya, clinics serving FSWs. Clinic staff informed clinic clients of the study objectives and participant selection criteria. Clients then voluntarily chose to participate. All participants were age 18 or older, residents of Nairobi and self-identified FSWs. To recruit SDCs, staff working with an SDC support group at the local public hospital in Nakuru, Kenya, discussed the study with potential participants who then voluntarily participated. All participants were age 18 or older, residents of Nakuru and self-identified as being in a sexual relationship with someone of the opposite HIV status. Participants for FGDs with adolescent and young women were recruited with the help of a pre-existing community advisory board (CAB) that was affiliated with the local South African research group who conducted the data collection activities. The CAB conducted community meetings to share information about the research with potential participants and the community at large. Participants for the FGDs with adolescent women were age 14 to 17; those in FGDs with young women were 18 to 24. After obtaining parental permission for potential participants under the age of 18, adolescents and young women expressing interest in the study were recruited by phone.

The risk profile of potential participants was the key determinant for recruitment. Aside from age (for adolescent and young women) and partnership status (for SDCs), other demographic factors were not examined.

The study protocol was approved in the United States by FHI 360's Protection of Human Subjects Committee; in Kenya by the Kenya Medical Research Institute; and in South Africa by the University of the Witwatersrand Human Research Ethics Committee and the eThekweni District and KwaZulu-Natal Provincial Departments of Health.

Written, informed consent was obtained from all participants age 18 and above. For participants age 14 to 17, informed consent was obtained from their parents or caregivers, and informed assent was obtained from the participants.

### Data collection

In Kenya, we conducted six FGDs – three in Nairobi with a total of 33 FSWs aged 21 to 37, and three in Nakuru with SDCs aged between 24 and 48 with a total of 32 participants (16 men, 16 women). In South Africa, we conducted four FGDs with a total of 36 participants. Two were with adolescent girls age 14 to 17, and two were with young women age 18 to 24. One FGD in each user group was with participants from informal settlements, and one with participants from formal settlements; this was a proxy for socioeconomic status, as residents of informal settlements tend to have a lower socioeconomic status than those of formal settlements. In the FGDs, we explored the potential user groups’ interest in and preferences for three different formulations of PrEP – oral PrEP, a vaginal gel and an injectable. The FGD guides comprised a combination of standard and population-specific questions.

Prior to the FGDs, all study participants were given information on PrEP products, including how HIV-negative individuals could use PrEP to avoid becoming infected with HIV, that PrEP products contain ARV-based drugs similar to the ones used to treat HIV-positive individuals, that PrEP products are still being researched, descriptions of the different formulations of PrEP and how they would be used, and how PrEP would likely offer partial protection from HIV infection.

Each FGD was conducted by two research assistants trained in research ethics and qualitative research methods, and fluent in English and the local language (isiZulu in South Africa, Kiswahili in Kenya). One research assistant served as the moderator and the other as the note taker. All FGDs were audio-recorded and simultaneously transcribed and translated into English as needed.

### Data analysis

Through a standard iterative process, we developed a codebook to structurally and thematically code the FGDs using QSR NVivo 9.0. Initially, two data analysts independently coded a sub-set of transcripts, and inter-coder agreement was assessed. This process was repeated until inter-coder agreement was reached for each set. Subsequently, each transcript was coded once by a given analyst, with the analysts consulting one another frequently to ensure that coding decisions were made jointly. The codebook was updated as new themes emerged. Once all data were coded, we generated code reports and conducted inductive thematic analysis to identify major trends and themes.

## Results

### Attitudes towards PrEP, including acceptability, interest and concerns

#### FSWs, Kenya

FSWs in Kenya expressed great interest in PrEP products. However, the majority appeared to assume initially that PrEP would provide 100% protection against HIV, and many were disappointed to learn that they would still need to use condoms to prevent HIV and other sexually transmitted infections (STIs). The majority of FSWs’ concerns regarding PrEP were related to its degree of effectiveness and to their preference for a PrEP product that could prevent both HIV and other STIs. They feared that some FSWs (and other users) would forgo condoms if PrEP became available, thus increasing the likelihood of transmitting HIV and STIs.

FSWs’ interest in PrEP was not only related to preventing HIV transmission, but also to helping them earn more money – because they would feel safer accepting more clients or would feel more comfortable having sex without using condoms. As one FSW described:

They [FSWs] will not have unprotected sex carelessly because as we have heard from some of us earlier on; they said that they usually have unprotected sex when they are offered more money like 3000 [Kenyan Shillings, about $35 US dollars] for sex without a condom or 300 [Kenyan Shillings, about $3.50 US dollars] for sex with a condom. PrEP will help in protecting them from getting HIV while making more money.

A few FSWs expressed concern about side effects, either generally or in relation to particular formulations. Most of these participants emphasized the need to help each woman identify the formulation appropriate for her.

#### SDCs, Kenya

SDCs in all FGDs expressed enthusiastic support for PrEP. We did not systematically survey the SDCs on whether they were currently using condoms consistently, although some mentioned that they were using condoms. However, the overwhelming consensus was that PrEP could, should, or will replace – rather than accompany – condoms as the primary form of HIV prevention within SDCs. Many respondents said they would not want to use PrEP and condoms at the same time.

SDCs also expressed great optimism and hope that PrEP would help reanimate sex lives and draw couples closer together by reducing or eliminating the need for condoms, which many participants described as diminishing sexual pleasure: *You see, we forgot having unprotected sex, so our love for each other will be revived* [with PrEP]. Another respondent explained, *We will accept them* [PrEP products] *as will clear the thoughts of using condoms and hence improve the relationship with my wife*. Participants frequently mentioned PrEP as a way to alleviate the stress associated with condom breaks.

Few SDCs had concerns regarding PrEP, but those that did worried about its safety or about the burden of daily-use formulations to the user.

#### Adolescent girls, South Africa

Adolescents were also interested in PrEP as protection from HIV, although a few participants appeared confused about whether PrEP would protect them from pregnancy as well. They found PrEP particularly appealing because it would eliminate concerns about being seen while obtaining condoms from clinics and because they felt PrEP products could be used privately. As one adolescent explained, *PrEP products … are much easier to use because you can just take a pill, unlike condoms because you are scared to get them from the clinics because you do not know who is watching you*. Some girls expressed hesitation in regards to PrEP, stating that they would be interested in using it only after seeing other girls use it.

#### Young women, South Africa

Young women were excited about and interested in using PrEP products to prevent HIV infection. One concern mentioned by several young women was that it might be difficult for women to use coitally dependent PrEP formulations while under the commonly reported influence of alcohol or drugs prior to sexual activity. Another concern was that it would be challenging to negotiate PrEP use with a partner or that young women simply would not use the product with trusted partners, similar to condom use patterns. One young woman explained that if a male partner *says he does not want to use* [PrEP] *today, you will not use it*.

Unclear on the correct timing of the use of different formations of PrEP, some young women were concerned that PrEP might be difficult to use in the midst of a sexual encounter: *Once your hormones are high, you forget […] both your hormones are already high. When are you going to insert the gel? When are you going to take the pills once you are in the middle of it?* A few women thought their peers would be more willing to use PrEP if they could use it covertly. No differences were identified between adolescent and young women from informal and formal settlements.

### Preferences for PrEP formulations

#### FSWs, Kenya

The majority of FSWs clearly preferred an injectable over other formulations. They liked that one dose would last for a prolonged period of time and would require little user intervention, unlike a pill that they must remember to take every day. In addition, injections were perceived as relatively private, making it less likely that others would know a woman was using the product. A few women described alcohol use as potentially interfering with their ability to take a daily pill but not posing a problem with injections, as this respondent explained:

*Ah okay, I would prefer the injection because it is private and you can use it without your client or partner knowing. Just like the family planning, injection most women who are using it, their partners are not aware. Secondly I personally love taking alcohol so with the PrEP injection I will be safe because I won't need to remember to take it daily like the oral pill or vaginal gel*.

Most FSWs were concerned that they might forget or that it would be burdensome to take a pill consistently. Several women were concerned about side effects associated with an oral product or interactions between pills and alcohol. A few women were worried that people would see them taking pills, whereas others liked the idea of taking a pill because it was daily, something they controlled and similar to contraceptive pills.

Nearly all FSWs reported using some form of vaginal lubricant in the past; however, there was little interest in PrEP gel. This was because women thought they might forget to apply it before and/or after sex and because clients might be against its use. As one FSW noted, *If asking a client to wear a condom is challenging then you can only imagine what they will do if they see you applying the vaginal gel before sex. It is crazy*.

Most FSWs said they would prefer to apply a gel before sex rather than afterwards; however, they saw problems with applying the gel at any point. For example, although the gel might help with lubrication before sex, some FSW noted that there would not always be time to apply it before sex and that FSWs who are drunk or high might forget to use it. Those who cited specific limitations with post-coital use most often noted that women were tired after sex and unlikely to remember to use it.

#### SDCs, Kenya

The majority of SDCs strongly preferred injectable PrEP because it required little user involvement and was long-lasting, in contrast to pills or gel. For example, one person said:

I also believe that the injection will be ideal because one won't be getting a jab every day, maybe it will be three or six months instead of every time you want to have sex, you either take the pills or apply the gel.

Another respondent said:

If it will be for three months I will know it is after every three months, it is better than the pills as one can forget to swallow, or maybe you did not have the pills with you because you travelled, so one will be at high risk.

Others said an injectable would be preferable because they did not like swallowing pills or taking medicine. Some individuals, however, said they would not use an injectable because they feared needles or the pain associated with injections.

Few individuals preferred pills to the other formulations; reasons for preferring pills included: liking the idea of an intermittent pill taken only when having intercourse, being accustomed to taking pills and disliking injections. Disadvantages of pills included a dislike of pills, having to remember to take them regularly and needing to remember to take them along when leaving the house or traveling.

Only three individuals commented on the gel formulation, but these three individuals thought it would be good because it would not be painful like an injection and would help men learn about the vagina. Fewer than half of the females in the SDCs in our focus groups reported ever using a vaginal lubricant or medicine. Among those who had used a vaginal product, the majority had used a vaginal medicine during pregnancy. SDCs overwhelmingly agreed that a PrEP gel should be applied before sexual intercourse, as this respondent commented: *I was feeling that the gel should be applied way before even starting to romance because I believe that it will be ready to fight with the virus*. Couples insisted that a gel provided before sexual intercourse would be more effective than one provided afterward. Couples noted that condoms are used before, not after sex, and they compared pre-coital use to “prevention” and post-coital use to a “cure.”

#### Adolescent women, South Africa

Adolescents did not express a clear preference for a particular PrEP formulation but noted some benefits and limitations of each one. Participants noted that users could forget to take all PrEP formulations. Adolescents felt that the process of obtaining pills could be more private than obtaining condoms, since pills in general might be taken for a variety of reasons. Additionally, whereas PrEP injections would require users to wait in a clinic and a gel would require privacy to insert, pills could be taken relatively privately at any time and place. Additionally, one adolescent noted that it would be easier to take a pill every day than try to predict when sex might happen. Some disadvantages associated with PrEP pills included difficulty swallowing them and concerns that they might cause urine to smell bad.

Adolescents’ concerns about injections included their potential to be painful, with some preferring an injection in the arm rather than the buttocks because they perceived it as less painful. A few adolescents also believed walking or sitting would be difficult after receiving an injection to the buttocks. Several girls were uncomfortable with having to remove their pants or skirts in order to receive the injection.

Only a few (four) adolescents commented on gel dosing. Half felt that it was better to use a gel daily in order to establish a routine. They also noted that daily use would limit situations in which girls wanted to have sex but did not have the gel with them. The other half thought a gel formulation would be better if used intermittently, either pre or post-coitally, especially for adolescents who did not frequently engage in sexual relations.

Participants debated over whether gel or pills were preferable. Advantages of gel included the perception that coitally dependent use was less burdensome than a daily pill and less painful than an injection. Disadvantages included the belief that predicting sex could be difficult, inserting a vaginal gel was unpleasant and required privacy, and a gel could have an unpleasant odour.

#### Young women, South Africa

Some young women preferred PrEP pills compared to other formulations because they believed pills were safer than injections and more private than gel. However, others felt it would be difficult to take a pill every day. Some young women preferred a coitally-dependent gel over pills because they thought it would be easier to remember to apply a gel. Still others preferred injections over a pill because they thought injections would be safer, longer lasting, more private and difficult to forget. One woman expressed concern that injections might not be effective in combination with alcohol and drugs.

Compared to adolescents, young women expressed less concern over receiving an injection in the buttocks. Some young women actually preferred that location, explaining that they were already accustomed to receiving contraceptive injections there. No differences were identified between adolescent and young women from informal and formal settlements.

## Discussion

This study contributes to existing literature on the acceptability of ARV-based PrEP formulations among beneficiary groups. All four potential target populations expressed strong interest in these products, similar to the favourable reactions identified in previous research among other key groups [7, 11–21]. Furthermore, our research introduces the concept of choice into work on the acceptability of HIV prevention methods. Although the importance of choice has been recognized and incorporated into contraceptive provision, it is an emerging idea in HIV prevention, most likely because condoms have been the only method of protection thus far. A range of new potential HIV prevention methods is in development, but there continues to be tension about which to develop and move forward – and our research suggests that no one method will meet all needs.

Although the SDCs in our study preferred the injectable formulation, their rationale for their support of the method is consistent with SDC participants of the Partners PrEP trial of oral tenofovir-only or oral Truvada (tenofovir combined with emtricitibine) in Uganda; SDCs from both studies viewed use of a PrEP product as a “way out” of the “discordance dilemma,” as defined in the Partners PrEP study, that is, that long-term condom use is unrealistic for SDCs (i.e. expensive, inconvenient, uncomfortable and incompatible with the desire to become pregnant), with preserving health and remaining in the relationship seemingly incompatible [19].

Although most women in the CAPRISA 004 trial testing 1% tenofovir gel found it acceptable [7], in our study, hypothetical use of a PrEP gel had mixed acceptability. In particular, some SDCs, FSWs and adolescent women found it unacceptable due to the inconvenience of pre- and post-coital application. In addition, FSWs cited clients’ potential disapproval of the gel as a disadvantage, suggesting that they may not seek or be able to use it covertly. In contrast, African, Indian and Peruvian FSWs have been found to support covert use of gels, viewing this as an advantage of the formulation [13, 22]. It has been suggested that for young adult women, men's passive acceptance of microbicide gel use may be more important than covert use [23]. Notably, adolescent participants in our study did not mention disclosure to partners as playing a role in their interest in PrEP gel or other PrEP formulations. However, we did not ask them if they were sexually active, and those who were not would not have had experience communicating with partners about HIV prevention or considering issues of disclosure.

This study provides some initial information on the preferences of at-risk populations for different formulations of PrEP, a topic about which little is yet known. In our study, most (but not all) FSWs and SDCs in Kenya preferred an injectable PrEP formulation, which they viewed as long-lasting and private. There was greater variation among adolescent and young women in South Africa regarding their preferences for PrEP formulation, with each group noting some benefits and limitations associated with each one. Factors affecting method preferences among the target groups included privacy of the method (for all but SDCs). Adolescent and young women especially were concerned with how PrEP products could be used privately; in particular inserting gels was seen as difficult to do privately while, conversely, pills were viewed as a product that could be used without others such as partners, friends, or family members noting or asking questions. Concerns over privacy are likely linked to an underlying stigma of adolescent and young women having sex, an issue that must be addressed to stimulate demand for PrEP products among this group.

Other factors that affected method preferences included: frequency of use, painfulness, the need for user intervention, side effects, interaction with alcohol and drugs, coital dependence, partner approval and convenience. Interestingly, efficacy was not mentioned as a factor influencing formulation preference, although it was mentioned in reference to the acceptability of formulations; however, it is possible that participants assumed all methods would be equally effective.

Offering PrEP users a choice of methods could also provide an additional benefit – the process of educating users on the methods available could serve as an opportunity to provide individualized risk-reduction counselling. PrEP trials routinely offer this and see benefits associated with the provision of this information. Additionally, method distribution and the regular HIV testing required for PrEP provision is another opportunity for the provision of brief counselling.

Among the limitations of our study are the small number of FGDs, the use of convenience sampling which may not have produced a fully representative sample of the target populations and questions about hypothetical use of these PrEP products, which may differ from both clinical trial use and real-world usage of available products. We also did not systematically collect information on whether condom migration would occur with the introduction of PrEP formulations, although some groups brought this up during the discussion. An additional limitation is that the context-specific nature of the results may limit generalizability of the findings to other cultures and country settings, but this ultimately relates to our main observation and conclusion.

## Conclusions

Women at risk of HIV in sub-Saharan Africa are a diverse population and do not necessarily share the same life circumstances, social norms, risk profiles or perceived ability to use particular PrEP formulations. Their circumstances and needs, as well as choices, are also likely to change over time as their lives take course, depending, for example, on age, relationship status and parity. Sub-Saharan African women's preferences for HIV prevention methods and formulations may be similar within particular user groups – or not, highlighting the importance of ensuring the availability of choice in HIV prevention methods.

## References

[1] Person AK, Hicks CB (2012). Pre-exposure prophylaxis – one more tool for HIV prevention. Curr HIV Res.

[2] Baeten J, Celum C (2012). Oral antiretroviral chemoprophylaxis: current status. Curr Opin HIV AIDS.

[3] Grant RM, Lama JR, Anderson PL, McMahan V, Liu AY, Vargas L (2010). Preexposure chemoprophylaxis for HIV prevention in men who have sex with men. N Engl J Med.

[4] Van Damme L, Corneli A, Ahmed K, Agot K, Lombaard J, Kapiga S (2012). Preexposure prophylaxis for HIV infection among African women. N Engl J Med.

[5] van der Straten A, van Damme L, Haberer JE, Bangsberg DR (2012). How well does PREP work? Unraveling the divergent results of PrEP trials for HIV prevention. AIDS.

[6] Marrazzo JM, Ramjee G, Nair G, Palanee T, Mkhize B, Nakabiito C Pre-exposure prophylaxis for HIV in women: daily oral tenofovir, oral tenofovir/emtricitabine or vaginal tenofovir gel in the VOICE study (MTN 003).

[7] Abdool Karim Q, Abdool Karim SS, Frohlich JA, Grobler AC, Baxter C, Mansoor LE (2010). Effectiveness and safety of tenofovir gel, an antiretroviral microbicide, for the prevention of HIV infection in women. Science.

[8] Ross J, Stover J (2013). Use of modern contraception increases when more methods become available: analysis of evidence from 1982–2009. Glob Health Sci Pract.

[9] Frost J, Darroch J (2008). Factors associated with contraceptive choice and inconsistent method use, United States, 2004. Perspect Sex Reprod Health.

[10] Gray A, Smit J, Manzini N, Beksinska M Systematic review of contraceptive medicines. “Does choice make a difference?”.

[11] Nodin N, Carballo-Dieguez A, Ventuneac AM, Balan IC, Remien R (2008). Knowledge and acceptability of alternative HIV prevention bio-medical products among MSM who bareback. AIDS Care.

[12] Heffron R, Ngure K, Mugo N, Celum C, Kurth A, Curran K (2012). Willingness of Kenyan HIV-1 serodiscordant couples to use antiretroviral based HIV-1 prevention strategies. J Acquir Immune Defic Syndr.

[13] Galea JT, Kinsler JJ, Salazar X, Lee SJ, Giron M, Sayles JN (2011). Acceptability of pre-exposure prophylaxis as an HIV prevention strategy: barriers and facilitators to pre-exposure prophylaxis uptake among at-risk Peruvian populations. Int J STD AIDS.

[14] Liu AY, Kittredge PV, Vittinghoff E, Raymond HF, Ahrens K, Matheson T (2008). Limited knowledge and use of HIV post- and pre-exposure prophylaxis among gay and bisexual men. J Acquir Immune Defic Syndr.

[15] Mimiaga MJ, Case P, Johnson CV, Safren SA, Mayer KH (2009). Preexposure antiretroviral prophylaxis attitudes in high-risk Boston area men who report having sex with men: limited knowledge and experience but potential for increased utilization after education. J Acquir Immune Defic Syndr.

[16] Golub SA, Kowalczyk W, Weinberger CL, Parsons JT (2010). Preexposure prophylaxis and predicted condom use among high-risk men who have sex with men. J Acquir Immune Defic Syndr.

[17] Dunkle K, Wingood G, Camp C, DiClemente R Intention to use pre-exposure prophylaxis among African-American and white women in the United States: results from a national telephone survey.

[18] Van der Elst EM, Mbogua J, Operario D, Mutua G, Kuo C, Mugo P (2013). High acceptability of HIV pre-exposure prophylaxis but challenges in adherence and use: qualitative insights from a phase I trial of intermittent and daily PrEP in at-risk populations in Kenya. AIDS Behav.

[19] Ware NC, Wyatt MA, Haberer JE, Baeten JM, Kintu A, Psaros C (2012). What's love got to do with it? Explaining adherence to oral antiretroviral pre-exposure prophylaxis for HIV-serodiscordant couples. J Acquir Immune Defic Syndr.

[20] Brooks RA, Landovitz RJ, Kaplan RL, Lieber E, Lee SJ, Barkley TW (2012). Sexual risk behaviors and acceptability of HIV pre-exposure prophylaxis among HIV-negative gay and bisexual men in serodiscordant relationships: a mixed methods study. AIDS Patient Care STDS.

[21] Terris-Prestholt F, Hanson K, MacPhail C, Vickerman P, Rees H, Watts C (2013). How much demand for New HIV prevention technologies can we really expect? Results from a discrete choice experiment in South Africa. PLoS One.

[22] Greene E, Batona G, Hallad J, Johnson S, Neema S, Tolley EE (2010). Acceptability and adherence of a candidate microbicide gel among high-risk women in Africa and India. Cult Health Sex.

[23] Rupp RE, Rosenthal SL (2003). Vaginal microbicides and teenagers. Curr Opin Obstet Gynecol.

